# Expression of vascular endothelial growth factor and pigment epithelial-derived factor in a rat model of retinopathy of prematurity

**Published:** 2011-06-10

**Authors:** John S. Hartmann, Hilary Thompson, Haibo Wang, Shami Kanekar, Wei Huang, Steven J. Budd, M. Elizabeth Hartnett

**Affiliations:** 1 University of North Carolina, Department of Ophthalmology, Chapel Hill, NC; 2 Moran Eye Center, University of Utah, Salt Lake City, UT; 3 School of Public Health, Louisiana State University, New Orleans, LA

## Abstract

Objective: To determine the effects of oxygen fluctuations on pigment epithelial-derived factor (PEDF) and vascular endothelial growth factor (VEGF)/PEDF ratios in a relevant rat model of retinopathy of prematurity (ROP).

**Methods:**

The expression of retinal *PEDF* mRNA and of VEGF and PEDF protein were determined using real-time polymerase chain reaction or enzyme-linked immunosorbent assays at different postnatal day ages for rat pups raised in room air (RA) or in a rat model mimicking ROP. Statistical outcomes were determined with factorial analyses of variance. Mean VEGF and PEDF protein levels were determined at different ages for rats in the ROP model and for RA-raised rats, and the ratio of VEGF/PEDF protein versus age was plotted. At postnatal day (P) 14, inner retinal plexus vascularization had extended to the ora serrata in pups raised in RA. In the ROP model, avascular retina persisted at P14 and intravitreous neovascularization developed at P18. Therefore, VEGF and PEDF expression was determined in the ROP model and in RA-raised rat pups at P14 and P18.

**Results:**

Older age was associated with increased *PEDF* mRNA (p<0.001), PEDF protein (p=0.005), and VEGF protein (p=0.005), and VEGF protein (p<0.0001). Exposure to fluctuations of oxygen in the 50/10 oxygen-induced retinopathy model compared to RA was associated with increased *PEDF* mRNA (p=0.0185), PEDF protein (p<0.0001), or VEGF protein (p<0.0001). The VEGF/PEDF ratio favored angiogenic inhibition (<1.0) before but not on P14, when avascular retina persisted in the ROP model but not in RA. The VEGF/PEDF ratio favored angiogenesis (>1.0) at P14 and P 18 when intravitreous neovascularization occurred in the ROP model.

**Conclusions:**

Increased expression levels of VEGF and PEDF are associated with older postnatal day age or with exposure to fluctuations in oxygen in the 50/10 oxygen-induced retinopathy model compared to RA. PEDF protein more closely associates with avascular retinal features and neovascularization than does VEGF protein or the VEGF/PEDF in the ROP model. Although PEDF has been proposed as a potential treatment in ROP, interventional studies using PEDF in an ROP model to potentially reduce intravitreous neovascularization are required to determine timing, efficacy, and dose of PEDF.

## Introduction

Retinopathy of prematurity (ROP), first reported in 1941 [1], reached epidemic status over the following decades, blinding many preterm infants. At that time, ROP was associated with high, unregulated oxygen delivery [2,3]. When inspired oxygen levels were reduced, ROP was virtually eliminated. Advances in neonatal care and technology have since led to increased survival of very low birthweight infants of young gestational age [4] and ROP has re-emerged [5]. High oxygen at birth is now avoided in countries that have implemented the technology to monitor and regulate oxygen and thus may not be a cause of most cases of ROP [6]. Rather, other factors have been associated with severe ROP, including poor postnatal weight gain [7] and oxygen stresses apart from high oxygen at birth, including fluctuations in transcutaneous oxygen levels [8]. Investigators have suggested that lower inspired oxygen concentrations and perhaps fewer fluctuations in oxygen are associated with the reduced incidence of ROP [9]. However, a recent large-scale prospective clinical trial compared 85%–89% oxygen saturation to 91%–95% oxygen saturation and found the lower oxygen saturation range to be associated with a reduced incidence of ROP, but also with increased infant mortality [10]. Therefore, more detailed investigation is needed regarding the effects of oxygen levels on severe ROP features and retinal development in preterm infants. Such knowledge would also aid in finding therapeutic agents for infant ROP.

Michaelson, Ashton, and Patz [2,3,11] developed a hypothesis about how severe ROP occurred. It was believed that high oxygen at birth led to avascular retinal areas that became hypoxic when infants were weaned to room air (RA). The retinal hypoxia stimulated the production of an angiogenic factor or factors [2,3,11]. Of numerous angiogenic factors studied since the early hypothesis, vascular endothelial growth factor (VEGF) has been recognized as one of the most important in retinal diseases [12]. VEGF is increased in models of oxygen-induced retinopathy (OIR) with hypoxia-induced angiogenesis following high constant oxygen-induced constriction [13] and in human ROP [14]. Excessive signaling of VEGF through VEGF receptor 2 (VEGFR2) has been found to disrupt developmental angiogenesis [15]. Conversely, inhibition of VEGF signaling through VEGFR2 has reduced pathologic intravitreous neovascularization [16-20] without interfering with ongoing retinal vascular development [16,18,19]. *VEGF* mRNA was detected in an infant retina that had severe ROP [21], and infants operated on for progressive stage 4 ROP had elevated ocular VEGF protein compared to control infants undergoing congenital cataract surgery [22]. Finally, intravitreous injections of anti-VEGF agents have been reported to reduce vascularly active features, such as intravitreous neovascularization in severe ROP [23]. However, later retinal detachment has been reported in some infants treated with anti-VEGF agents [24] Concerns also exist about using anti-VEGF agents in preterm infants because VEGF is involved in angiogenesis and neuroprotection, both important in developing preterm infants. Therefore, other treatments must be considered.

One such treatment is pigment epithelial-derived factor (PEDF), which is a potent angiogenic inhibitor [25]. Exogenous administration of PEDF decreased neovascularization in OIR models of hypoxia-induced angiogenesis following high constant oxygen-induced capillary constriction [26,27] or in models of laser-induced injury [28]. In some studies, decreased levels of PEDF have been associated with proliferative diabetic retinopathy and age-related macular degeneration (AMD) [29,30], whereas increased levels have been shown to reduce morbidity in neovascular AMD [31]. PEDF is believed to regulate angiogenesis through multiple mechanisms, including downregulation of VEGF expression [32]. A ratio of VEGF/PEDF of less than 1.0 has been associated with reduced angiogenesis in some models of OIR [33-35]. However, these models may not reflect the severe ROP that occurs in infants in neonatal units that monitor and regulate oxygen for preterm infants. We became interested in PEDF as a potential treatment in ROP because it is neuroprotective [25] and anti-inflammatory [36], potentially may regulate VEGF-induced pathologic angiogenesis, and has been safely tolerated in a human clinical trial [31].

Much of what has been learned about proposed mechanisms in ROP has been discovered using models of high oxygen to induce capillary constriction followed by relative hypoxia-induced angiogenesis [34,37,38]. These models of OIR with hypoxia-induced angiogenesis following high constant oxygen [13] may be relevant to human ROP described in the 1940s that was associated with high unregulated oxygen [39]. We use a model developed by John Penn [40], in which Sprague-Dawley pups and their dams are placed into a controlled environment where the oxygen fluctuates between 50% and 10%. The model uses oxygen levels relevant to ROP in that it yields rat arterial oxygen concentrations similar to transcutaneous oxygen levels reported in infants with severe ROP [40]. The model also produces a characteristic appearance of severe ROP with peripheral avascular retina similar to zone II ROP [40,41], followed by retinal tortuosity similar to plus disease [42], and then intravitreous neovascularization at the junctions of vascular and avascular retina, similar to stage 3 ROP [41,43]. The model uses fluctuations in oxygen, a risk factor for severe ROP [8]. Further, abnormalities in photoreceptor and post-receptor responses in electroretinograms of pups exposed to this model share similarities with those found in human preterm infants with severe ROP [44]. For these reasons, the Penn 50/10 model is described as the ROP model [42]. We and others have used this model to study the effects of oxygen and other stresses on the development of severe ROP features [18,19] and the relationships between features and angiogenic growth factor signaling pathways [45-49]. In this study, as a first, step to determine whether PEDF might be a therapeutic agent to safely prevent severe ROP, we sought to determine the effect of relevant oxygen levels on the regulation of VEGF and PEDF in association with severe ROP features using the Penn rat model of ROP.

## Methods

### ROP model

All animal studies were approved by the University of North Carolina’s Institute for Laboratory Animal Research (Guide for the Care and Use of Laboratory Animals) and the University of Utah’s Institute of Animal Care and Use Committee, and complied with the ARVO Statement for the Use of Animals in Ophthalmic and Visual Research. Within 4 h of birth at postnatal day (P) 0, litters of 16 newborn Sprague-Dawley rat pups with their dams (Charles River, Wilmington, MA) were placed in an Oxycycler incubator (Biospherix, New York, NY) to cycle inspired oxygen between 50% oxygen and 10% oxygen every 24 h. Pups from other litters were used to supplement deficient litters. To maintain the reproducibility of the ROP model, litters were never depleted below 12 pups. Often, this required that whole litters be used for individual postnatal day measurements. After seven cycles of oxygen fluctuations at P14, pups were then placed into RA for 4 days [40]. Oxygen levels were monitored daily and recalibrated as needed. Carbon dioxide in the cage was also monitored daily and was flushed from the system by maintaining sufficient gas flow and by adding soda lime, if needed.

We analyzed retinas from pups of several postnatal day ages preceding and including P14 when full vascularization of the inner retinal plexus occurred in RA-raised pups, but persistent avascular retina was present in the ROP model. We also analyzed pups at P18 when intravitreous neovascularization occurred in the ROP model. Postnatal day 0 was analyzed as the baseline value. In the ROP model, P8, P12, and P14 occurred immediately following a hypoxic session (10% inspired O_2_) and P11 and P13 following a hyperoxic session (50% inspired O_2_). Following P14, pups were placed into an RA environment (21% FiO_2_).

### Dissection of retinal tissue for mRNA and protein analyses

Animals were euthanized with pentobarbital (80 mg/kg intraperitoneal injection) at the beginning of the change in inspired oxygen level. Eyes were placed into phosphate buffered saline (PBS) after the hyaloid vessels and remaining vitreous were removed. For fresh tissue, retinas were dissected without ora serratas. The retina was removed from the eyecup and frozen in modified radioimmunoprecipitation assay (RIPA) buffer (20 mM Tris base, 120 mM NaCl, 1% Triton X-100, 0.5% sodium deoxycholate, 0.1% sodium dodecyl sulfate (SDS), and 10% glycerol) with a protease inhibitor cocktail (1:100; Sigma, St. Louis, MO) and 1 M orthovanadate (1:100; Sigma), then stored at −80 °C for protein or in RNA later (Applied Biosystems, Carlsbad, CA) for RNA until analysis.

### Dissection of retinal tissue for retinal flat mounts

For flat mounts, following enucleation, eyes were fixed in 2% paraformaldehyde for 2 h. Retinas were isolated as described previously [50], but with ora serratas intact, and placed into PBS after the hyaloid vessels and remaining vitreous were removed. By making four incisions 90 degrees apart, the retinas were flattened and then placed onto microscope slides.

### Tissue staining and analysis of flat mounts

To stain the vasculature, the flattened retinas were first permeabilized in ice-cold 70% v/v ethanol for 20 min, then in PBS/1% Triton X-100 for 30 min, and then incubated with Alexa Fluor 568 conjugated *G. simplicifolia* (Bandeiraea) isolectin B4 (5 μg/ml; Molecular Probes, Carlsbad, CA) in PBS overnight at 4 °C, as previously described [18]. Images of the retinal blood vessels were captured using a Nikon 80i Research Upright Microscope, Melville, NY with Surveyor/Turboscan software and digitally stored for analysis.

### Real time quantitative PCR

Total RNA was extracted using the RNAeasy Mini Kit (Qiagen, Valencia CA). Assays were performed using the Applied Biosystems 7500 Real-Time PCR System. Briefly, 1 μg of total RNA was reverse-transcribed into cDNA using a High Capacity cDNA Kit (Applied Biosystems), according to the manufacturer’s protocol. Each TaqMan reaction (16 μl) contained 20 ng of cDNA, 8 μl of TaqMan PCR MasterMix (Applied Biosystems) and 1 μM forward primer, 1 μM reverse primer, and 1 μM probe. Primers were specific for rat (annealing temperature 60 °C) *PEDF*: forward 5′-CCA ACT CTT TGC AGG ACA TG-3′, reverse 5′-TCA CAG GTT TGC CGG TAA TC-3′, and probe 5′ -ACA GTC CTT GTT TGA GTC CCC TGA C −3′. Primers and probes were made by UNCs Oligonucleotide Core Facility. Each sample was run in duplicate, and each experiment included three non-template control wells. The 7500 System Software calculates cycle threshold (Ct) automatically for each well and each value was normalized to rat β-actin (*Actb*). Rat *Actb* was chosen as the control gene because its expression had previously been found to be stable under various oxygen stresses. Primers for rat *Actb* were forward 5′-TGC CTG ACG GTC AGG TCA-3‘, reverse 5′-CAG GAA GGA AGG CTG GAA G-3‘, and probe 5′-CAC TAT CGG CAA TGA GCG GTT CCG-3‘. Postnatal day age 0 indicated retinal tissue harvested within 4 h of birth and was used in analysis for both the ROP and RA groups. For graphical representation of *PEDF* mRNA, this value was scaled to 1.0 and used as the standard against which fold changes in mRNA expression were compared. However, for statistical analysis, raw data were used as described below.

### Protein analysis

Retinal samples, which were stored in RIPA buffer at −80 °C, were thawed, homogenized, and centrifuged (16,000× g, 10 min, 4 °C). Total protein was quantified with a Bicinchoninic Acid (BCA) Protein Assay Kit (Bio-Rad, Hercules, CA, modified from the Lowry assay) [51]. Supernatants were assayed without dilution in duplicate using commercially available ELISA kits, raised against rat VEGF (R&D Systems, Rochester MN) or human PEDF (BioProducts, Middletown, MD). The VEGF ELISA measures all VEGF splice variants, with the most prevalent splice variant, VEGF_164_, representing the greatest percentage of the ELISA value [52]. The mean minimum detectable dose for VEGF was 6.4 pg/mL. For the PEDF ELISA, the manufacturer estimates a 10–50× reduced affinity for rat PEDF; however, our results met criteria for test validity and fell within the standard curve. To take into account potential reduced affinity, we compared retinal values from rat pups in the 50/10 OIR to rat pups in control RA taken at the same postnatal day ages.

### Calculation of VEGF/PEDF ratios

For each postnatal day age, the mean values of VEGF or PEDF protein determined for the ROP model were normalized to the respective mean RA values of growth factors at the same postnatal day ages. These normalized values were then used to form a VEGF to PEDF ratio under the two conditions.

### In situ hybridization

Whole eyes were fresh frozen in Optimal Cutting Temperature (OCT) compound and stored at −80 °C. Serial 14-µm-thick sections were cut for in situ hybridization, which was performed at the University of North Carolina’s In Situ Hybridization Core Facility. Mouse *PEDF* cDNA [53] (1374 bp, GenBank AF017055) was kindly supplied by Joshua Barnett (Vanderbilt University, Nashville, TN). *PEDF* cDNA was subcloned into the pBluescript SK vector (Stratagene, La Jolla, CA) and linearized with HindIII and XbaI to produce templates for in vitro transcription of antisense and sense riboprobes. This transcription was performed using a DIG RNA Labeling Kit (Roche Diagnostics, Indianapolis, IN) to produce a single stranded digoxigenin-11-uridine-triphosphate-labeled probe. Hybridization with a sense strand riboprobe was performed under identical conditions to a negative control. A digoxigenin probe was localized in tissue using an alkaline-phosphatase-conjugated anti-digoxigenin antibody and visualized using NBT/BCIP solution (Roche Diagnostics, Mannheim Germany). PEDF in situ hybridization was imaged using an Olympus DSU-IX81 spinning disc confocal microscope.

### Immunostaining for PEDF

Immunostaining was done using mouse anti-PEDF (Millipore, Temecula CA) as primary antibody and visualized using Alexa-Fluor 588-conjugated goat anti-mouse secondary antibodies (Invitrogen, Carlsbad CA) using an Olympus (Tokyo, Japan) DSU-IX81 spinning disc confocal microscope. No primary antibody was used in the controls.

### Statistical analysis

For each postnatal day age, at least five retinas from different pups taken from at least two different litters were analyzed. The mean fold changes relative to beta-actin are graphically represented, with error bars representing standard errors. While these normalized ratios were used for graphical representation in the figures, to avoid bias, the raw data were used for statistical analysis as described below.

Initially, *Actb* was analyzed by regression analysis of the ratio of each *Actb*/*VEGF* splice variant or *PEDF* mRNA for the Ct1 value against respective *Actb*/*VEGF* splice variants or *PEDF* mRNA for the Ct2 value, and the slope was found to be indistinguishable from 1.0, the ideal value [54]. The geometric means of the product of the two ratios were then determined for each postnatal day age and treatment, and this was the outcome analyzed. For the analysis of VEGF and PEDF protein by ELISA, the protein concentration was the outcome analyzed. A factorial ANOVA with a completely randomized treatment arrangement was used to determine the significance of the factors, postnatal day age, and treatment (RA versus 50/10 OIR model). Post-hoc testing of the treatment and increasing postnatal day age by treatment interaction means was accomplished using protected *t*-tests on least-squares means, with alpha level adjustment for multiple comparisons among means accomplished using a method of simulation [55].

## Results

### ROP model

**Figure 1:**
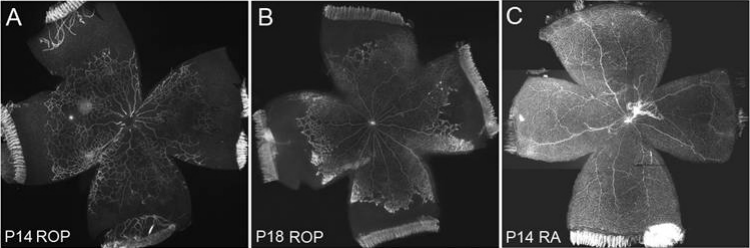
Retinal flat mounts at P14 and P18 in the ROP model and at P18 in RA samples. Representative lectin-stained flat mounts from rat pups in the ROP model or RA conditions. In the ROP model, avascular retina remains at P14 (**A**), while in RA, intraretinal vascularization is complete at P14 (**C**). In the ROP model, after seven cycles of oxygen fluctuations by P14, pups are then moved into RA for 4 days. At P18, intravitreous neovascularization occurs at the junction of vascularized and avascular retina (**B**).

Figure 1 shows representative flat mounts of retinas from an RA-raised pup at P14 (Figure 1C), and from pups in the ROP model at P14 (Figure 1A) and P18 (Figure 1B). In the ROP model at P14, avascular retina comprised 32.8±10.6% of the total retinal area, whereas in RA pups, there was no avascular retina present [52]. At P18, avascular retina comprised 23.1±6.2% of the total retina and intravitreous neovascularization was present in approximately eight clock hours of all retinas in the ROP model [52], making up 2.29±0.68% of the total retinal area. Numbers are mean±SEM.

### PEDF tissue localization

**Figure 2:**
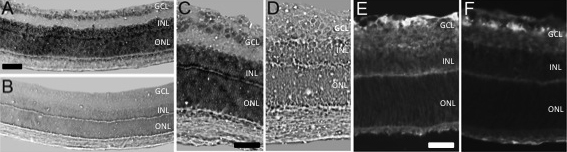
Tissue labeling of *PEDF* mRNA and protein in rat ROP model at P14. Sections of P14 rat retina in ROP model labeled by in situ hybridization for *PEDF* mRNA showing message in the ganglion cell layer (GCL), and in the inner and outer nuclear layers (INL, ONL; **A**, **C**). Control using sense probe showed no signal (**B**, **D**). Immunostaining for PEDF protein in P14 ROP model showed labeling in the inner and outer plexiform layers and inner and outer nuclear layers (**E**). No primary control showed nonspecific staining in the ganglion cell layer (**F**). Magnification calibration bars are 100 µm (**A**, **B**) and 50 µm (**C**-**F**).

To determine the localization of *PEDF* mRNA within the retina of the ROP model, we first performed in situ hybridization on P14 retinal sections from pups in the ROP model. At this postnatal day age, retinal vascularization is restricted to 70%–75% of the retina in the ROP model [52], as shown in Figure 1. *PEDF* mRNA was detected in ganglion cells and cells throughout the inner and outer nuclear layers (Figure 2). Sense labeled sections of P14 ROP retinas were blank (Figure 2).

We then localized PEDF protein in retinal cryosections of P14 pups in the ROP model and found labeling in inner and outer plexiform layers and in the inner nuclear layer. A lower level of PEDF immunostaining was also seen in the outer nuclear layer (Figure 2E). Figure 2F shows nonspecific staining of the secondary antibody.

### PEDF mRNA quantification

**Figure 3:**
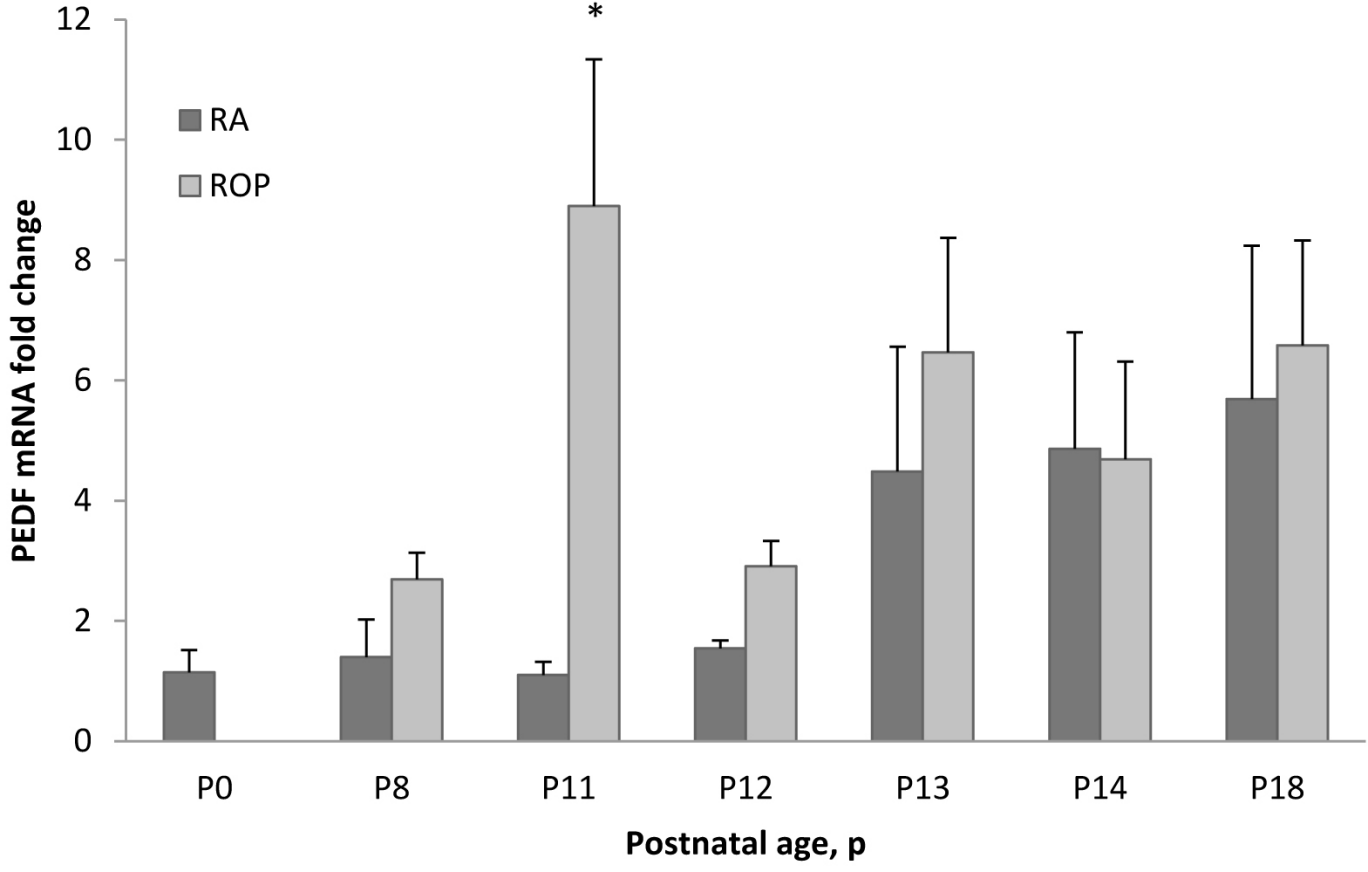
Quantification of *PEDF* mRNA in ROP model at different postnatal day ages. Real time polymerase chain reaction values for PEDF expression in the retina of rat pups from selected postnatal days (P) 0 through P18 in RA or the ROP model. Increased PEDF expression associated with older developmental age (p=0.0001) or with the ROP model compared to RA (ANOVA, p=0.0185). *Post-hoc testing revealed increased *PEDF* mRNA at P11 in the ROP model compared to RA samples (p=0.0017). Error bars represent standard errors. Each time point had at least five retinas from different pups taken from at least two different litters. mRNA indicates mRNA.

To quantify the expression of *PEDF* mRNA, real time PCR was performed on retinal lysates of RA-raised pups and those in the ROP model at postnatal day ages between P0 and P18. The relative *PEDF* mRNA level was similar for the P0 and P8 time points and increased from P11 to P18 in rats raised in RA (Figure 3). In the ROP model, retinal *PEDF* expression was higher compared to RA counterparts at most time points and appeared to be expressed to a greater extent than in RA pups at earlier postnatal day ages. Within the model, the pattern of *PEDF* mRNA expression level fluctuated in association with oxygen concentration in the ROP model. It tended to increase following hyperoxia at P11 and P13 and decrease following hypoxia at P8, P12, and P14. Based on statistical analysis, increased fold expression of *PEDF* was significantly associated with older postnatal day age (ANOVA, p=0.0001) or with the ROP model compared to RA (ANOVA, p=0.0185). Post-hoc testing revealed only a significantly increased fold expression in *PEDF* mRNA at P11 in the ROP model compared to the RA group (p=0.0017).

### PEDF protein concentration in RA and ROP models

**Figure 4:**
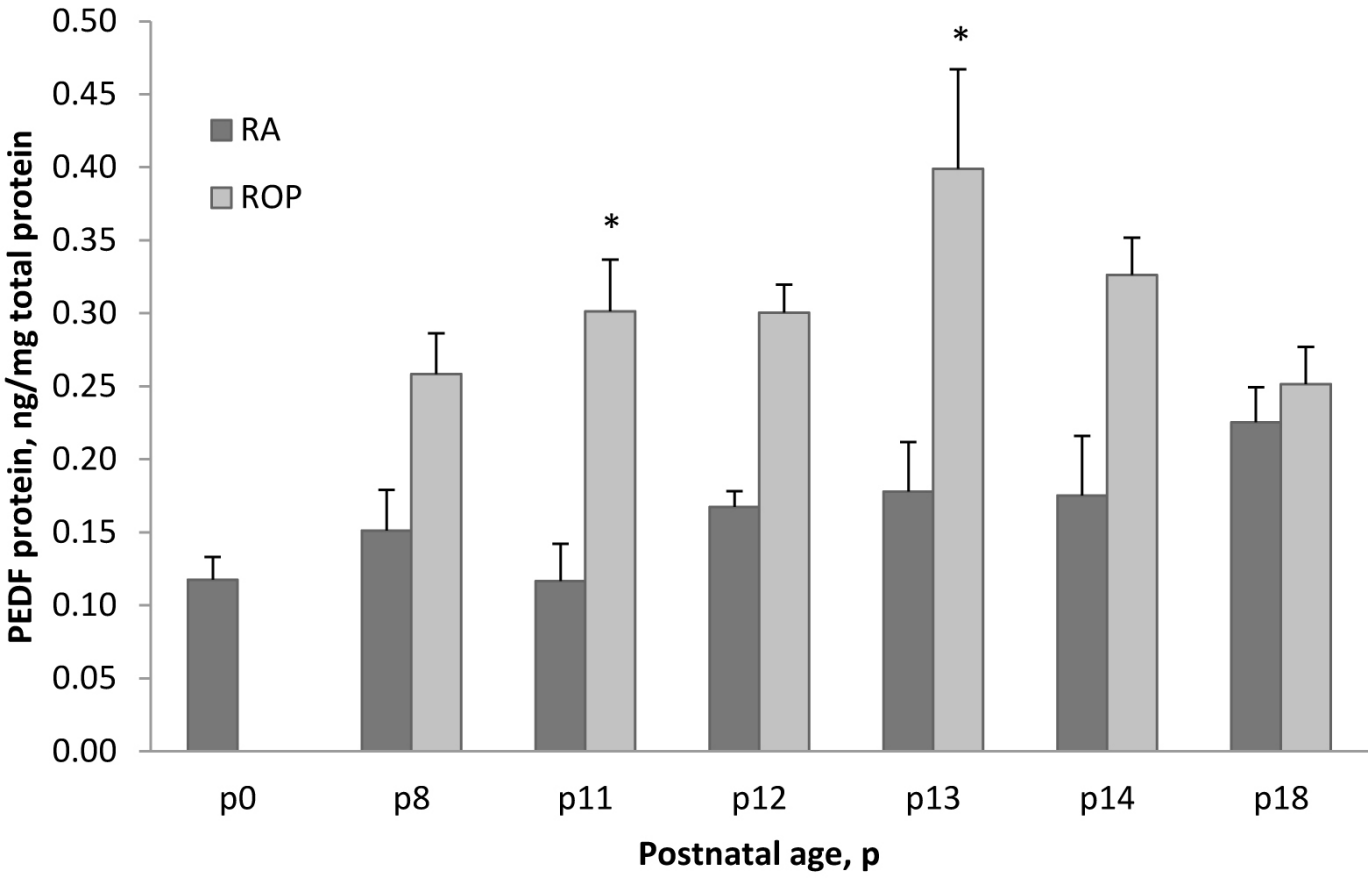
Quantification of PEDF protein in ROP model at different postnatal day ages. ELISA measurements of retinal PEDF protein from selected postnatal days (P) 0 through P18 in RA or in the ROP model. Increased PEDF associated with older developmental age (p=0.005) or the ROP model compared to RA samples (ANOVA, p<0.0001). *Post-hoc testing revealed increased PEDF in the ROP model compared to RA at P11 and P13 (p=0.0041, p=0.0022 respectively). Error bars represent standard errors. Each time point had at least five retinas from different pups taken from at least two different litters.

PEDF protein was measured in retinal lysates taken at the same postnatal day ages as mRNA from rats raised in RA or exposed to the ROP model. PEDF protein increased with older postnatal day age in retinas from RA-raised rats (Figure 4). For rats in the ROP model compared to those raised in RA, the pattern of PEDF increased before and on P14 when avascular retina persisted in the ROP model, but not in RA samples. However, at P18 when neovascularization was present in the ROP model, the protein levels of PEDF and RA samples were similar. Compared to that with *PEDF* mRNA, the pattern of PEDF protein concentration was less closely associated with changes in oxygen concentration in the model. Statistical analysis revealed increased PEDF in association with older developmental age (ANOVA, p=0.005) or with exposure to the ROP model compared to RA samples (ANOVA, p<0.0001). Based on post-hoc testing, PEDF was significantly increased in the ROP model compared to RA samples at P11 and P13, each following a 24 h exposure to hyperoxia (p=0.0041, p=0.0022, respectively).

### VEGF protein concentration in RA samples and in the ROP model

**Figure 5:**
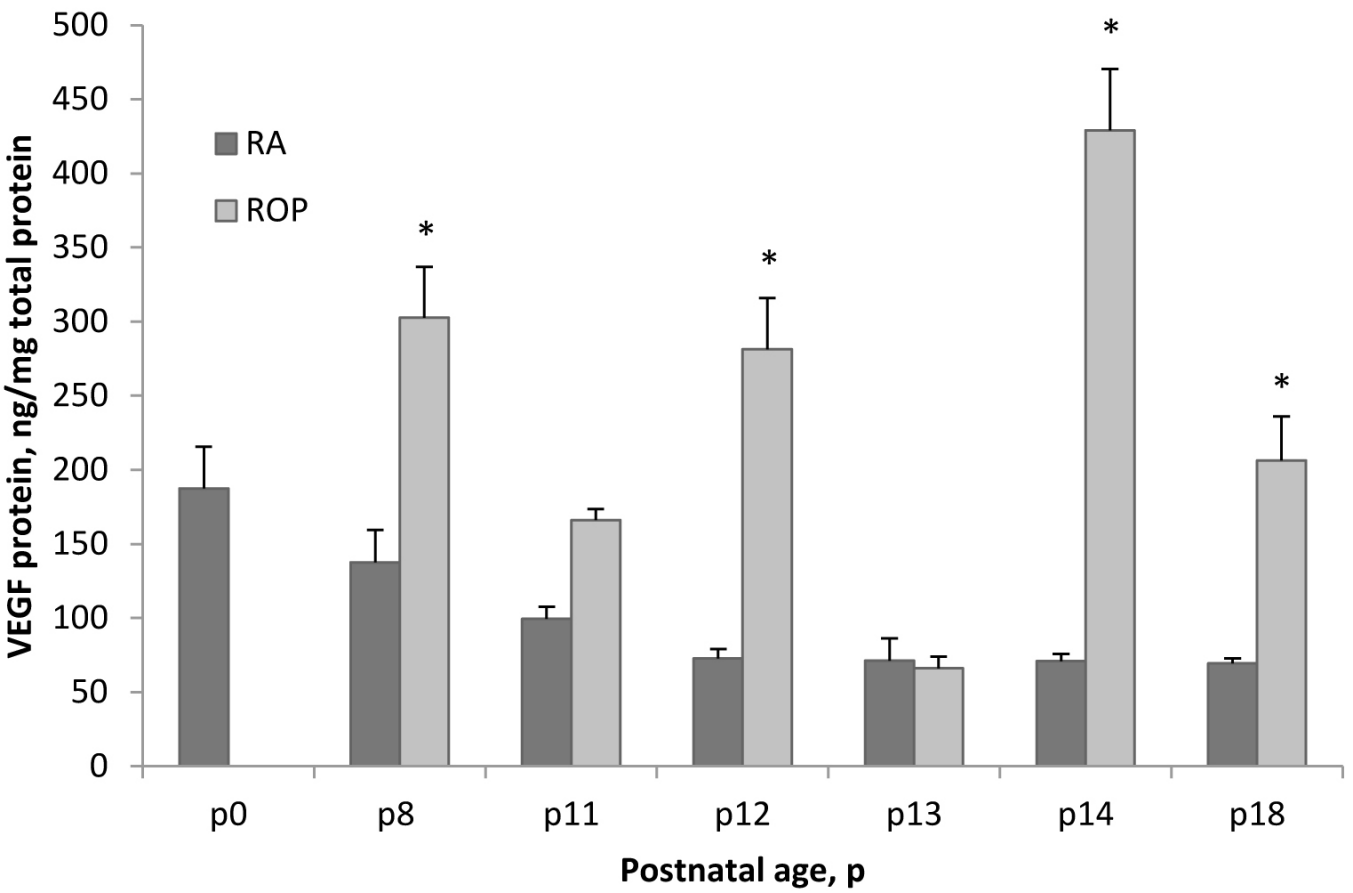
Quantification of VEGF protein in ROP model at different postnatal day ages. ELISA measurements of retinal VEGF protein from postnatal days (P) 0 through P18 in RA or the ROP model. Increased VEGF associated with older developmental age (ANOVA, p<0.0001) or the ROP model compared to RA samples (ANOVA, p<0.0001). Post-hoc testing revealed increased VEGF protein in the ROP model compared to RA at P8, P12, P14, and P18 (p=0.0002, p<.0001, p<0.0001, p=0.0117). In the ROP model, VEGF was significantly greater following hypoxic compared to hyperoxic cycles (P12 versus P11 [p=0.0014], P12 versus P13, and P14 versus P13; p<0.0001, each). Error bars represent standard errors. Each postnatal day age had at least five retinas from different pups taken from at least two different litters.

According to previously published studies, the VEGF_164_ isoform is the most prevalent during RA retinal development and in the ROP model [52,56]. It was also shown that *VEGF* mRNA and protein increased with older postnatal day age and in the ROP model [56]. In agreement with this, VEGF protein (Figure 5) was significantly increased in association with older postnatal day age (ANOVA, p<0.0001) or with exposure to the ROP model compared to RA samples (ANOVA, p<0.0001). Based on post-hoc testing, there were significant increases in VEGF protein in the ROP model compared to RA samples at P8, P12, P14, and P18 (p=0.0002, p<0.0001, p<0.0001, p=0.0117). In the ROP model, VEGF was significantly greater following hypoxic compared to hyperoxic cycles (P12 versus P11 [p=0.0014], P12 versus P13, and P14 versus P13; p<0.0001, each).

### VEGF/PEDF ratios

**Figure 6:**
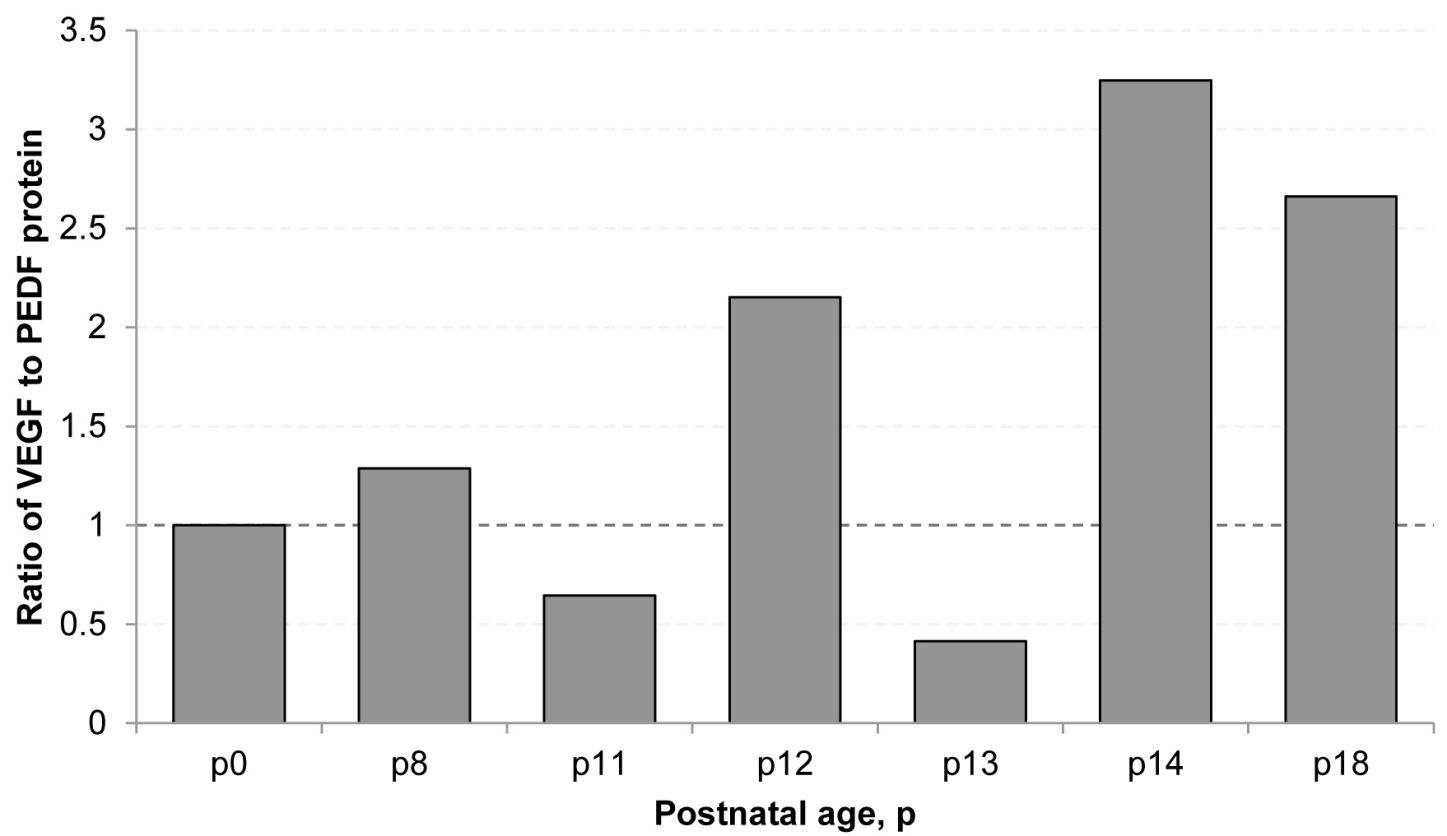
Ratio of VEGF/PEDF protein in ROP model normalized to RA values. The ratio of mean VEGF/PEDF in the ROP model normalized to respective RA means at selected postnatal day ages. A ratio >1.0 favors angiogenesis (P8, P12, P14, P18) and a ratio <1.0 favors angiogenic inhibition (P11 and P13).

To compare VEGF and PEDF, we first developed ratios of the ROP to RA mean retinal protein values for VEGF and PEDF at each postnatal day age tested. We then created a ratio between VEGF and PEDF at each postnatal day age and graphically represented these (Figure 6). This is similar to methods used in previous reports [35]. At P14, retinal vascularization of the inner plexus is complete in RA controls, whereas avascular retina persists in the ROP model [52] (Figure 1). At P11 and P13, the VEGF/PEDF ratio favored angiogenic inhibition (ratio less than 1.0). However, at P14, the VEGF/PEDF protein ratio favored angiogenic stimulation (greater than 1.0). The ratios were approximately 3.0 at P14 and P18 in the ROP model.

## Discussion

Normal development of the retinal vasculature depends on the integration of several signaling events influenced by a delicate balance of angiogenic agonists and inhibitors. Of many factors, two important ones in retinal development are VEGF [57,58] and PEDF [59]. VEGF is an angiogenic agonist, upregulated in hypoxia [60], whereas PEDF is an angiogenic antagonist, upregulated by hyperoxia [59]. Using a model of ROP, we studied the effects of oxygen stresses, relevant to preterm infants with ROP, on PEDF expression and the relative differences in the VEGF/PEDF ratio in comparison to control rat pups raised in RA. Compared to RA, we found that retinal PEDF was increased in association with avascular retina in the ROP model.

The interaction of VEGF and PEDF has been explored in other models. Oxidative stress increased the angiogenic potential of cultured RPE cells in association with an increased fold expression of VEGF to PEDF [33]. Using a model of high oxygen-induced retinopathy (OIR) in rats, the fold expression of VEGF was reduced during hyperoxia but increased during relative hypoxia-induced endothelial proliferation [35]. Previously, using the ROP model, PEDF was found to approach levels of that in RA samples at P18 [61], and the VEGF/PEDF ratio favored angiogenesis in association with the development of intravitreous neovascularization in the ROP model at P18. We had similar findings in our study. However, we also studied younger postnatal day ages before the development of intravitreous neovascularization at P18. Earlier ages in the model may relate to the preterm infant retina before the development of severe ROP. We found that the pattern of the VEGF/PEDF ratio of retina in the ROP model normalized to RA was similar to that of VEGF protein, favoring angiogenic stimulation following a hypoxic cycle (10% O2) and favoring inhibition following a hyperoxic cycle (50% O2). The VEGF/PEDF ratio favored angiogenic inhibition before but not at P14, when avascular retina persisted in the ROP model but vascularization of the inner plexus was complete in RA pup retinas. The VEGF/PEDF ratio, PEDF, and VEGF protein favored angiogenesis at P18 when intravitreous neovascularization occurred. The PEDF protein level appeared more closely associated with features of the ROP model than did VEGF or the VEGF/PEDF ratio.

Most reports that studied young postnatal day ages in models of OIR did not use relevant animal models such as the Penn ROP model. Gao et al. [62] examined the effect of high constant oxygen in rats and found that the ratio of VEGF/PEDF corresponded to the degree of intravitreous neovascularization. The investigators also found a difference according to the strain of rat, in that Brown Norway rats had more severe neovascularization than did Sprague-Dawley rats, suggesting a genetic component to oxygen sensitivity. Based on a verbal communication, no differences in the extent of intravitreous neovascularization were found between the two rat strains subjected to oxygen fluctuations. However, it is not clear what the extremes in inspired oxygen were.

PEDF might be considered as a therapy to prevent severe ROP in human preterm infants, potentially as an intravitreal injection like anti-VEGF agents currently used for adults [23]. The reasons to consider PEDF are that it inhibits angiogenesis [27], in part by promoting apoptosis [26,63], it mitigates inflammation, and it is neuroprotective [64]. Therefore, it would potentially be safer than anti-VEGF agents. In addition, it was reported that overexpression of PEDF with an adenovirus associated vector was not shown to adversely affect the radial expansion of retinal vessels in wild type mice raised in RA, suggesting that retinal vascular development can proceed in conditions of overexpressed PEDF [65]. Additional evidence of potential protective effects of PEDF was shown in *PEDF*^−/−^ mice that were found to be more susceptible to hyperoxia-mediated retinal vascular constriction and retraction compared to *PEDF*^+/+^ mice subjected to the same conditions. This occurred even though retinal VEGF protein was increased in *PEDF*^−/−^ compared to *PEDF*^+/+^ [66]. These data suggest the presence of PEDF may even benefit the development of intraretinal vascularization, at least in the condition of high oxygen.

The findings in this manuscript are associations and are not causative, and further study is warranted to address the question of whether exogenous PEDF delivered early when the VEGF/PEDF ratio is less than 1.0 would lead to persistence of the avascular retina, an unwanted outcome in ROP. Our findings may be relevant to ROP currently because the Penn ROP model more closely reflects oxygen levels and the appearance of ROP in current neonatal units than earlier models of OIR that were developed to mimic the ROP of the 1940s.

Changes in the patterns of PEDF concentrations based on the exposure to hypoxia or hyperoxia were best appreciated in fold expressions of mRNA, and less so in protein. As anticipated [59], *PEDF* mRNA was upregulated following periods of hyperoxia at P11 and P13. The PEDF antibody recognizes human PEDF but is estimated by the manufacturer to have a lower affinity for rat PEDF. For this reason, we normalized mean values of PEDF and VEGF protein to respective RA means. PEDF protein expression may also be less sensitive to fluctuating oxygen levels than mRNA because of the time required for protein translation, post-translational modification, and possibly sequestration of growth factors in extracellular matrix.

In summary, we found that compared to RA counterparts, both VEGF and PEDF were significantly increased in the rat ROP model at early and late postnatal day ages. However, we found the relative expression level of VEGF to PEDF protein favored angiogenic inhibition before but not at P14 when avascular retina persisted in the model but vascularization of the inner retinal plexus was complete in RA. Evidence suggests that PEDF can be released in response to several stresses and have beneficial effects as a neuroprotectant and anti-inflammatory agent. Further study is needed to determine the timing and type of delivery of PEDF in the ROP model. In this study, the relative expression of PEDF protein was more closely associated with early features in the ROP model, i.e., avascular retina, than was the VEGF or the VEGF/PEDF ratio.
